# Gonorrhœal Infection of the Mouth in New-Born Infants

**Published:** 1892-12

**Authors:** 


GONORRHŒAL INFECTION OF THE MUCOUS MEMBRANE OF THE MOUTH OF NEW-BORN INFANTS.—Rosinsky (*Zeitschrift fur Geburtshalfe und Gynakologie*, Band XXII, Heft 1 und 2), from the study of five cases of gonorrhœal infection of the mouth, in the Konigsberg obstetrical clinic, has drawn the following picture of the disease: Without preceding inflammatory redness, a white discoloration appears upon the anterior two-thirds of the tongue, the plaques of Bednar, the hamulous pterygoidews and along the ligamentum pterygomandibularum in the lower jaw, finally upon the front part of the gums. After 24 to 36 hours, the color becomes yellow. The patches elevate themselves plateau-like over the surrounding tissues, and their surfaces are raw. The superficial epithelium forms, with extravisated pus cells, a thick layer resembling the scrapings from the cut surface of a septic spleen. On the third day, the regeneration of the epithelium begins; this is marked by an inflammatory redness around the edge of the patch. Healing follows without treatment in an ideal manner, no trace of scar or discoloration remaining. From the microscopical examination of some excised tissue, Rosinsky has gleaned the following: The gonococci were never found in stained sections, intracellular. They were seldom found intracellular in the superficial flakes. Gonococci cannot penetrate into the body of healthy living cells ; they accomplish this only when the single cells are cut off from the conditions of life. In the connective tissue, gonococci invasion was found. Rosinsky believes this to be typical for pure gonorrhœal inflammation of the mucous membrane of the mouth in adults in contradistinction to infants, he believes due to the tenderness of the epithelium of the mouth in the new-born.—*Annals of Gynecology and Pœdiatry.*

